# Diagnostic Accuracy of Global Pharma Health Fund Minilab™ in Assessing Pharmacopoeial Quality of Antimicrobials

**DOI:** 10.4269/ajtmh.17-0289

**Published:** 2017-11-06

**Authors:** Hui Pan, William Ba-Thein

**Affiliations:** 1 Shantou-Oxford Clinical Research Unit, Shantou University Medical College, Shantou, Guangdong, People’s Republic of China;; 2 Department of Microbiology and Immunology, Shantou University Medical College, Shantou, Guangdong, People’s Republic of China

## Abstract

Global Pharma Health Fund (GPHF) Minilab™, a semi-quantitative thin-layer chromatography (TLC)–based commercially available test kit, is widely used in drug quality surveillance globally, but its diagnostic accuracy is unclear. We investigated the diagnostic accuracy of Minilab system for antimicrobials, using high-performance liquid chromatography (HPLC) as reference standard. Following the Minilab protocols and the Pharmacopoeia of the People’s Republic of China protocols, Minilab-TLC and HPLC were used to test five common antimicrobials (506 batches) for relative concentration of active pharmaceutical ingredients. The prevalence of poor-quality antimicrobials determined, respectively, by Minilab TLC and HPLC was amoxicillin (0% versus 14.9%), azithromycin (0% versus 17.4%), cefuroxime axetil (14.3% versus 0%), levofloxacin (0% versus 3.0%), and metronidazole (0% versus 38.0%). The Minilab TLC had false-positive and false-negative detection rates of 2.6% (13/506) and 15.2% (77/506) accordingly, resulting in the following test characteristics: sensitivity 0%, specificity 97.0%, positive predictive value 0, negative predictive value 0.8, positive likelihood ratio 0, negative likelihood ratio 1.0, diagnostic odds ratio 0, and adjusted diagnostic odds ratio 0.2. This study demonstrates unsatisfying diagnostic accuracy of Minilab system in screening poor-quality antimicrobials of common use. Using Minilab as a stand-alone system for monitoring drug quality should be reconsidered.

## INTRODUCTION

Poor-quality drugs are a serious public health problem worldwide. According to the World Health Organization (WHO),^1^ poor quality drugs include substandard antimicrobials, which are authorized medical products failing to meet either their quality standards and/or specifications, and falsified antimicrobials, which deliberately/fraudulently misrepresent their identity, composition, or source. The prevalence of poor-quality antimicrobials varies from 0% to more than 50% globally, and their consequences as treatment failure, drug resistance, adverse drug events, and even death have been frequently reported.^2^

Drug quality surveillance involves screening and confirmatory testing. Although screening tests are less accurate than confirmatory tests, they are preferred over more expensive confirmatory tests such as high-performance liquid chromatography (HPLC) for field testing, especially in resource-limited low- and middle-income countries.^3–5^ There are common screening tools, such as WHO Checklist, paper chromatography cards, PharmaCheck, near infrared spectrometry, Counterfeit Device #3, Raman spectrometry, and Global Pharma Health Fund (GPHF) Minilab™.^4,5^

GPHF Minilab™ is a semi-quantitative thin-layer chromatography (TLC)–based commercially available test kit made by the GPHF, Merck Darmstadt, Germany. It has been widely used in drug-quality monitoring in as many as 95 countries, most of them low- or middle-income.^6^ More than 800 GPHF-Minilab units have been supplied globally until 2017.^6,7^ Its use has been supported by the WHO and the United States Pharmacopeia Drug Quality Information program.^6,7^ In light of its widespread use, even as the reference test to evaluate the diagnostic accuracy of a handheld Raman spectrometer,^8^ several studies have investigated the performance reliability of the Minilab.^7,9–15^ Most of these studies focused mainly on anti-malarial drugs^9,10,12,14,15^ and few studies on other antimicrobials.^13,15^ Based on the limited sample sizes, some studies reported the Minilab as a tool that can identify falsified, but not substandard, medicines.^9–13,15^

Being the second country only after India in producing poor-quality antimicrobials,^2,16^ China is in need of reinforcing drug-quality control by using cost-effective screening tests for its heavy surveillance workload. In this study, we examined the Minilab’s applicability in screening/monitoring (specifically, its diagnostic accuracy) for five common antimicrobials (amoxicillin, azithromycin, cefuroxime axetil, levofloxacin, and metronidazole) which are usually prescribed for infectious diseases caused by gram-positive and gram-negative bacteria, mycoplasma, *Chlamydia*, and anaerobes. Anti-malarial drugs, the most commonly investigated antimicrobials by the GPHF Minilab™ system in literature, were not included in this study, because malaria is not endemic in the study region. HPLC was used as the reference standard confirmatory test because it is recommended by different pharmacopeias including the Pharmacopoeia of the People’s Republic of China (PPRC) and widely used by the China Food and Drug Administration.

## MATERIALS AND METHODS

**Figure 1.**
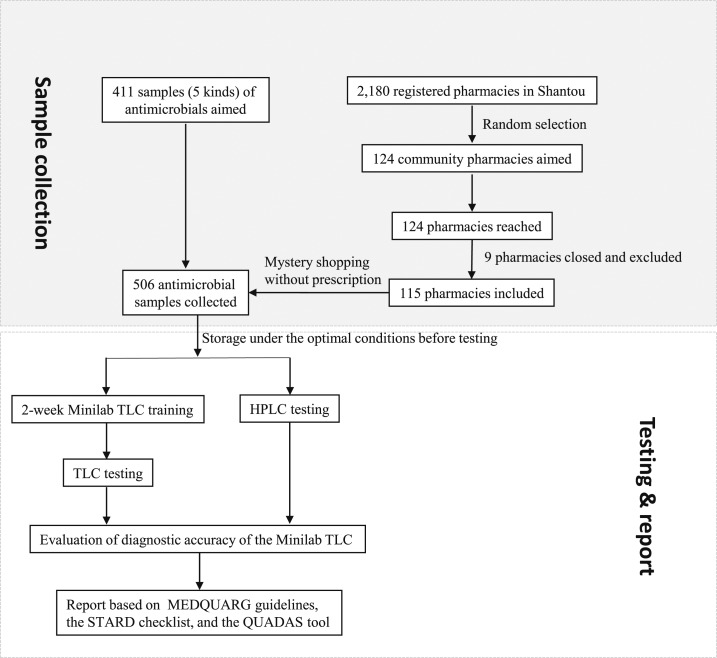
Flowchart outlining the procedure of sample collection, testing, and reporting the quality of common antimicrobials and the diagnostic accuracy of the Minilab. HPLC = high-performance liquid chromatography; TLC = thin-layer chromatography.

The STARD flowchart of the study is shown in Figure 1. Five kinds of antimicrobials in 506 batches were purchased from community pharmacies in Shantou, Guangdong, China, in this cross-sectional study (May to July 2013) as described previously.^3^ The selection criteria^3^ for these antimicrobials were as follows: 1) frequently used in hospitals and pharmacies^17^; 2) easily destroyed under suboptimal storage conditions^2,17^; 3) commonly reported to have no active ingredient^2,17^; 4) listed in the National Essential Drug List of China.^3,17^

With the estimated prevalence (15%) of poor-quality drugs in developing countries, the sample size of antimicrobials was calculated using the formula previously described^18,19^ as follows: *n* = confidence level_2_ * estimated prevalence * (1 − estimated prevalence) * design effect * (1 + non-response error)/margin of error_2_ = 1.96_2_ × 0.15 × (1 − 0.15) × 2 × (1 + 0.05)/0.05_2_ = 411.

From 2,180 pharmacies registered in Shantou, 124 pharmacies were randomly selected and visited based on their geographic distributions, nine were found to be closed during the sampling period, and 115 were finally included in the study. Following the consumer purchase behavior in China, our study staff and student volunteers approached the target pharmacies like patient’s relatives (mystery shoppers) and purchased the target antimicrobials without any prescription and following the pharmacy staff’s recommendation. Totally, 506 batches of antimicrobials (out of the estimated sample size as 411) were collected and transported to our laboratory in the same day and stored at room temperature (15–25°C) and relative humidity level of 60% or lower before testing. GPHF Minilab™ kits and the reference standard antimicrobials (all in tablet formulation) used for Minilab tests were purchased from the GPHF, Germany. Following the Minilab protocols, TLC was used to test drug samples in triplicate for the relative concentration of active pharmaceutical ingredients (APIs). Two HPLC-experienced operators received a 2-week Minilab TLC training, twice the standard training period recommended by the device’s manufacturer.^20^ In our proficiency testing, the operators achieved 100% agreement with the preset standard and substandard samples, nearly twice the rate achieved in a standard 1-week training.^21^ Reference standard antimicrobials for HPLC tests were purchased from Huipeng Science and Technology, China (http://www.szhuipeng.cn/), one of the authorized suppliers for the National Institutes for Food and Drug Control, China. Testing samples with HPLC was described previously.^3^ Minilab TLC and HPLC tests were performed simultaneously, while the operators were blinded to the results. All tests were done in two independent laboratories (the Shantou-Oxford Clinical Research Unit and the Bio-analytical Laboratory) of Shantou University Medical College.

For HPLC, “out of range” (i.e., tested positive) was defined as an API outside the acceptable range 93–107% for metronidazole or 90–110% for all other drugs (Table 1). For the Minilab, “out of range” was defined as an API of less than 80% of the pharmacopoeial standards for all drugs. The diagnostic test characteristics of Minilab TLC test were measured (i.e., sensitivity [Sn], specificity [Sp], positive predictive value [PPV], negative predictive value [NPV], positive likelihood ratio [PLR], negative likelihood ratio [NLR], diagnostic odds ratio [DOR],^22^ and adjusted diagnostic odds ratio [ADOR] with 95% confidence intervals [95% CI]). ADOR was calculated by adding 0.5 to all original counts as some of them were zero.^23^ All the analyses were done through SPSS statistics V17.0 and SAS 9.4. Our results were reported according to the MEDQUARG guidelines,^24^ the STARD checklist,^25^ and the QUADAS tool^26^ where possible.

**Table 1:** The active pharmaceutical ingredients (API) of antimicrobials (*N* = 506)

Antimicrobial (*N* = 506)	Actual content of API* mean (SD)	Range* (minimum–maximum)	Lower and upper quartiles	PPRC standard
Amoxicillin (*N* = 114)	94.1% (5.1)	74.3–122.0%	91.8–96.6%	90.0–110.0%
Azithromycin (*N* = 92)	101.3% (7.9)	84.5–122.9%	96.9–102.6%	90.0–110.0%
Cefuroxime axetil (*N* = 91)	97.9% (2.9)	90.4–103.9%	95.8–100.3%	90.0–110.0%
Levofloxacin (*N* = 101)	98.7% (4.1)	83.5–110.2%	96.1–101.5%	90.0–110.0%
Metronidazole (*N* = 108)	93.4% (1.8)	84.1–98.1%	92.1–94.5%	93.0–107.0%

PPRC = Pharmacopoeia of the People’s Republic of China (2010); SD = standard deviation.

* Compared with reference standard antimicrobials by high-performance liquid chromatography.

## RESULTS

Our results are summarized in Tables 1 and 2. Of 506 batches of antimicrobial samples, 2.6% (13/506) did not meet the standard limits of the Minilab TLC system, but all of them (13/13) were within the expected quality standards of the PPRC in the HPLC system. This false positive detection occurred exclusively with cefuroxime axetil, accounting for 14.3% of cefuroxime axetil samples (13/91). On the other hand, false negativity was seen in 15.2% of samples (77/506) that met the quality standard limits in the Minilab TLC but failed in the HPLC system. Twenty-two percent (17/77) of the poor-quality samples were higher than the upper standard limit of the PPRC. Accordingly, the characteristics of Minilab TLC test were recorded as follows: Sn 0% (95% CI: 0– 4.7%); Sp 97.0% (95% CI: 94.9–98.4%); PPV 0; NPV 0.8 (95% CI: 0.8–0.9); PLR 0; NLR 1.0 (95% CI: 1.0–1.0); DOR 0; and ADOR 0.2 (95% CI: 0.01–3.4).

**Table 2:** Diagnostic accuracy of GPHF Minilab™ tests in the surveillance of poor-quality antimicrobials (*N* = 506)

Antimicrobial	Minilab TLC	HPLC		Sn (95% CI) (%)	Sp (95% CI) (%)	PPV (95% CI)	NPV (95% CI)	PLR (95% CI)	NLR (95% CI)	DOR (95% CI)	ADOR* (95% CI)
		Positive (*n*)	Negative (*n*)								
Amoxicillin (*N* = 114)	Positive	0 (TP)	0 (FP)	0 (0–19.5)	100.0 (96.3–100.0)	NaN†	0.9 (0.8–0.9)	NaN†	1.0 (1.0–1.0)	NaN†	5.6 (0.1–290.2)
	Negative	17 (FN)	97 (TN)								
Azithromycin (*N* = 92)	Positive	0 (TP)	0 (FP)	0 (0–20.6)	100.0 (95.3–100.0)	NaN†	0.8 (0.7–0.9)	NaN†	1.0 (1.0–1.0)	NaN†	4.6 (0.1–242.2)
	Negative	16 (FN)	76 (TN)								
Cefuroxime axetil (*N* = 91)	Positive	0 (TP)	13 (FP)	NaN†	85.7 (76.8–92.2)	0	1.0 (1.0–1.0)	0	1.2 (NaN†)	NaN†	5.8 (0.1–305.7)
	Negative	0 (FN)	78 (TN)								
Levofloxacin (*N* = 101)	Positive	0 (TP)	0 (FP)	0 (0–70.8)	100.0 (96.3–100.0)	NaN†	1.0 (0.9–1.0)	NaN†	1.0 (1.0–1.0)	NaN†	28.1 (0.5–1635.3)
	Negative	3 (FN)	98 (TN)								
Metronidazole (*N* = 108)	Positive	0 (TP)	0 (FP)	0 (0–8.6)	100.0 (94.6–100.0)	NaN†	0.6 (0.5–0.7)	NaN†	1.0 (1.0–1.0)	NaN†	1.6 (0.03–83.5)
	Negative	41 (FN)	67 (TN)								
Total (*N* = 506)	Positive	0 (TP)	13 (FP)	0 (0–4.7)	97.0 (94.9–98.4)	0	0.8 (0.8–0.9)	0	1.0 (1.0–1.0)	0 (NaN†)	0.2 (0.01–3.4)
	Negative	77 (FN)	416 (TN)								

ADOR = adjusted DOR; DOR = diagnostic odds ratio; FN = false negative; FP = false positive; HPLC = high-performance liquid chromatography; NLR = negative likelihood ratio; NPV = negative predictive value; PLR = positive likelihood ratio; Positive = failing TLC or HPLC; Negative = passing TLC or HPLC; PPV = positive predictive value; Sn = sensitivity; Sp = specificity; TLC = thin-layer chromatography; TN = true negative; TP = true positive.

* ADOR was calculated by adding 0.5 to all counts in table (see Materials and Methods for details).

† NaN = not a number.

The prevalence of poor-quality samples determined, respectively, by Minilab TLC and HPLC was amoxicillin (0% versus 14.9%), azithromycin (0% versus 17.4%), cefuroxime axetil (14.3% versus 0%), levofloxacin (0% versus 3.0%), and metronidazole (0% versus 38.0%).

## DISCUSSION

The prevalence of poor-quality antimicrobials as determined by the Minilab system varies by country and by drug from 0% to 26%.^9–13,27,28^ For example, the prevalence of poor-quality amoxicillin was between 0% and 10% in Tanzania, Ghana, Nigeria, and the United Kingdom.^13,27^

This wide range of reported prevalence most likely originates from the subjectivity and skills required in operating the Minilab system and its diagnostic accuracy that varies with drugs. Highly variable Minilab test outcomes have been reportedly due to the operator’s visual estimation of TLC and operational skills for manually spotting on the chromatographic plate.^15,21,29^ In a Minilab proficiency test conducted by the Tanzania Food and Drugs Authority, only five of nine experienced inspectors were able to correctly identify the API levels of antimicrobials.^21^ Drug-dependent variation in the diagnostic accuracy of the Minilab has been exemplified by its Sn as 0% for amoxicillin and 14% for co-trimoxazole, compared with HPLC-photodiode array detection.^13^ The WHO QAMSA study performed in six countries found that the Minilab tests could only identify 32% of the poor-quality drugs.^7^ The performance of the Minilab in literature is summarized in Supplemental Table 1.

The diagnostic accuracy of the Minilab seems to be independent from the prevalence of poor-quality antimicrobials in this study, because the Sn (0% for nearly all antimicrobials) and Sp (range: 85.7–100%) of the Minilab were not correlated with the prevalence between 0% and 38% by HPLC. The rationale behind this could be that the APIs of most samples were higher than 80% API threshold that the Minilab is designed to detect. The Minilab did not, in fact, fail to detect most poor-quality drugs in this study by the standards that the manufacturer set for the device, but it regarded the drugs having > 80% APIs as good quality, except the 13 cefuroxime axetil samples. Only one sample, that is, amoxicillin (API: 74.3% by HPLC) fell below the 80% API cutoff.

This highlights the importance of considering and evaluating the performance characteristics and indications of the Minilab TLC properly before use. Whereas HPLC could be less affordable or even unavailable in developing countries or regions with limited resources, the Minilab system is much cheaper (e.g., the cost of consumables for Minilab TLC versus HPLC: approximately 70 versus 300 RMB per sample in this study; 1 USD = 6.6 RMB), portable, and rather efficient for drug quality surveillance.^4,5,15^ As a semi-quantitative test, the Minilab performs well in detecting falsified drugs which contain no API or wrong API.^10–15,27^ For this reason, it has been broadly used as a screening test globally, especially in Vietnam, Nigeria, Tanzania, Congo-Kinshasa, Ghana, Laos, Myanmar, Ethiopia, Indonesia, and Colombia.^30^ However, this study confirms that the Minilab performs very poorly in detecting drug levels that are close to, but fall short of, pharmacopoeial standards. In addition, there is no upper-detection limit in the Minilab system; therefore, it failed to identify drugs with higher APIs, further reducing our estimates of its Sn. Along with others’ findings,^10–15,27^ a relatively high false-negative rate (15.2%) of detecting poor-quality antimicrobials with the Minilab TLC in this study suggests that had other studies^9,28^ used more sensitive and specific methods such as HPLC, near-infrared spectrometry, or Raman spectrometry, they would have yielded higher estimates of poor-quality drugs.

The three-level approach involving the Minilab system has been used by the WHO QAMSA and USP-PQM studies^7,31,32^ to monitor drug quality in limited-resource countries: 1) visual and physical inspections of the drug and its packaging; 2) rapid screening with GPHF Minilab™; 3) confirmatory testing. All samples failing in the screening test and a random selection of the samples passing the screening test are further tested by pharmacopeial methods for confirmation.^7,31,32^ This approach is helpful for detecting most falsified drugs and a certain proportion of substandard drugs in a large amount of samples^15^; however, it has limitations,^31^ such as leading to inconclusive results and failing to identify all substandard medicines. As proven by this study, this approach might not be applicable in countries such as China, where strict law enforcement requires conclusive identification of both falsified and substandard medicines.

Low Sn of the Minilab in detecting substandard medicines with either low or high APIs has negative influences at clinical level. To the extent that pharmacopoeial standards and dose formulations correctly reflect the therapeutic efficacy of a medicine, the medicines that have lower APIs threaten patient welfare and may also promote antimicrobial resistance.^33^ On the other hand, the medicines with higher APIs (comprising 22% of poor-quality samples in this study) which the Minilab falsely regard as good quality have a potential to cause adverse drug events. On the basis of these notions, we would recommend reconsidering the Minilab manuals for medicines with higher APIs and caution against the use of the Minilab as a stand-alone system for monitoring drug quality.

Sn and Sp used in previous studies^7,13^ can only partially indicate the performance of the Minilab. Its other test characteristics, such as PPV, NPV, PLR, NLR, DOR, or ADOR, are not available in literature, and therefore, the test accuracy of the Minilab system has been unclear. This study has demonstrated unsatisfying test characteristics of the Minilab—PPV (0), PLR (0), NLR (1.0), DOR (0), and ADOR (0.2)—in screening poor-quality antimicrobials. To better understand the test characteristics of the Minilab system on different medicines, further studies utilizing comprehensive sampling strategy and reliable confirmatory tests are needed.

In summary, this study demonstrates unsatisfying diagnostic accuracy of the Minilab system in screening poor-quality antimicrobials of common use. Using the Minilab as a stand-alone system for monitoring drug quality should, therefore, be reconsidered.

## Supplementary Material

Supplemental Table.
