# Supplementary material for: Diagnostic Accuracy of Global Pharma Health Fund Minilab™ in Assessing Pharmacopoeial Quality of Antimicrobials
Source: Am J Trop Med Hyg. 2017 Nov 6;98(1):344–8. doi: 10.4269/ajtmh.17-0289 (PMC5928700; doi:10.4269/ajtmh.17-0289)
Supplement: Supplementary file 1 [file tpmd170289.SD1.pdf]

Supplemental Table 1

Studies on surveillance of poor-quality medicines using the GPHF Minilab™

Reference (year)	Country	Study period	Sample size	Category of drug sampled	Sampling/testing strategies	Detection method	Results (including diagnostic accuracy)	Characteristics of falsified/ substandard drug
Visser et al. (2015)[9]	Gabon	2014–2015	432	16 kinds of anti-malarial	Pseudo-patients collected drugs from randomly selected pharmacies. All samples were tested by the Minilab, and those that failed were tested by HPLC-UV-PDA.	GPHF Minilab (TLC and disintegration tests) and HPLC-UV-PDA	Two of 432 samples (0.5%) failed the TLC test, in which one was falsified (no API), and the other was substandard (nearly half the dose) in the HPLC test. One (0.4%) of 266 samples failed the disintegration test. Compared with HPLC, the Sn and Sp of Minilab TLC for these two samples were both 100% (2/2).	No API, low APIs, expired medicines
Vijaykadga et al. (2006)[10]	Thailand	2003–2004	369	Anti-malarial (artesunate, chloroquine, mefloquine, quinine, sulfadoxine/ pyrimethamine, and tetracycline)	All registered private drugstores, groceries, government malaria clinics, and government hospitals were visited. Samples were screened by the Minilab TLC and disintegration tests at sentinel site; 10% of passed, 100% of failed and doubtful samples were confirmed by HPLC and disintegration tests at national laboratory.	GPHF Minilab (TLC and disintegration tests) and HPLC	All 369 (100%) samples passed the Minilab TLC test, but 15 (4.1%) of them failed the disintegration test at sentinel site. From further confirmation of 79 samples, two (2.5%) failed the HPLC test, and seven (8.9%) failed the disintegration test at national laboratory.	Low APIs
Bate et al. (2009)[11]	Ghana, India, Kenya, Nigeria, Tanzania, and Uganda	2008	78	Anti-malarial (amodiaquine, artemether-lumefantrine fixed-dose combination, artemether, artesunate, chloroquine, dihydroartemisinin, mefloquine, and sulfadoxine-pyrimethamine), antibiotics (erythromycin and ciprofloxacin), and antimycobacterial drugs (isoniazid and rifampicin)	Samples were obtained from the manufacturers or from the GPHF. Samples were screened by the Minilab and further tested by Raman spectrometry and NIR spectrometry tests.	GPHF Minilab (TLC and disintegration tests), Raman spectrometry, and NIR spectrometry	15%, 13%, 41%, and 47% of tested samples failed TLC, disintegration test, NIR spectrometry, and Raman spectrometry, respectively.	Not specified

(continued)

Supplemental Table 1
Continued

Reference (year)	Country	Study period	Sample size	Category of drug sampled	Sampling/testing strategies	Detection method	Results (including diagnostic accuracy)	Characteristics of falsified/substandard drug
Risha et al. (2008)[27]	Tanzania	2003–2005 (three phases)	1,257	Anti-malarial (quinine, artesunate, and sulfadoxine/pyrimethamine preparations), antibiotics (ampicillin, amoxicillin, metronidazole, ciprofloxacin, cotrimoxazole, and erthromycin), and antiretroviral drugs (lamivudine, zidovudine, indinavir, nevirapine, stavudine, didanosine, and their combinations)	Samples collected at key ports of entry and post-marketing surveillance were screened by the Minilab, and those that failed the screening tests or needed further dissolution testing were tested by full pharmacopoeial monograph testing.	GPHF Minilab (TLC and disintegration tests) and full pharmacopoeial monograph testing	Five of 1,257 (0.3%) failed the Minilab tests (all counterfeit). 46 of 1,257 (3.7%) samples failed the pharmacopoeial monograph tests (substandard).	Counterfeit and substandard
Lalani et al. (2015)[12]	Afghanistan	2009	7,740	Anti-malarial (chloroquine, sulfadoxine/pyrimethamine, artesunate, artemether, quinine, and primaquine)	Samples were covertly collected from randomly selected pharmacies. One hundred thirty-four samples were screened by the Minilab, of which 40 were tested by HPLC-UV-PDA and dissolution tests.	GPHF Minilab (TLC and disintegration tests), HPLC-UV-PDA, and dissolution test	All 134 (100%) samples passed visual inspection, 100% of 132 samples passed the Minilab TLC test, 33 (26%) of 126 samples failed the disintegration test. All 40 samples tested by HPLC passed the test, but 12 (32%) of 37 samples failed the dissolution test.	Substandard
Fadeyi et al. (2015)[13]	Ghana, Nigeria, and United Kingdom	N/A	35	Amoxicillin and co-trimoxazole	Samples were obtained from drug outlets, including pharmacies, licensed chemical stores, and drug vendors in Ghana, Nigeria, and United Kingdom using a convenience sampling method with an overt approach. All samples were tested by the Minilab, HPLC-PDA, and dissolution tests.	GPHF Minilab (TLC and colorimetric tests), HPLC-UV-PDA, and dissolution test	20 (100%), 18 (90%), 19 (95%), and 20 (100%) of 20 amoxicillin samples passed colorimetric, TLC, HPLC, and dissolution tests, respectively. 15 (100%), 13 (86.7%), 1 (6.67%), and 6 (40%) of 15 co-trimoxazole samples passed colorimetric, TLC, HPLC, and dissolution tests, respectively. Compared with HPLC, the Sn and Sp of Minilab TLC were 0% and 89.5% for amoxicillin, and 14% and 100% for co-trimoxazole, respectively.	Low APIs

(continued)

Supplemental Table 1

Continued

Reference (year)	Country	Study period	Sample size	Category of drug sampled	Sampling/testing strategies	Detection method	Results (including diagnostic accuracy)	Characteristics of falsified/ substandard drug
Bate and Hess (2010)[14]	Ghana and Nigeria	2007–2010	339	Anti-malarial	Samples were purchased covertly from randomly selected pharmacies. 339 samples were tested in the Minilab, of which 300 were further tested by Raman spectrometry	GPHF Minilab (TLC and disintegration tests), and Raman spectrometry	32 (9.4%) and 64 (18.9%) of 339 samples failed the visual inspection and the Minilab tests (TLC and disintegration tests), respectively. 29 (9.7%), 55 (18.3%), and 79 (26.3%) of 300 samples failed the visual inspection, the Minilab tests, and Raman spectrometry test, respectively.	Not specified
Khuluza et al. (2017)[15]	Malawi	2014–2015	155	Anti-malarial (artemether/ lumefantrine, sulfadoxine/ pyrimethamine, quinine hydrochloride, artesunate/ amodiaquine, quinine sulfate, dihydroartemisinin/ piperaquine) and antibiotics (phenoxymethylpenicillin, amoxicillin, ciprofloxacin, amoxicillin/clavulanic acid, chloramphenicol, and cefuroxime axetil)	Samples were overtly or covertly collected from randomly selected health facilities. All 155 samples were tested by the Minilab TLC and disintegration tests; 10 samples that failed in the Minilab and a random selection of 46 passed samples (56 in total) were further tested after pharmacopeial monographs.	GPHF Minilab (TLC and disintegration tests) and pharmacopeial monographs (dissolution test and assay)	Of 155 samples, five (3.2%) and four (2.6%) failed in visual inspection and the Minilab TLC/disintegration tests, respectively. Of 56 samples, 5 (8.9%), 4 (7.1%), and 7 (12.5%) failed in visual inspection, the Minilab TLC/disintegration tests, and dissolution test/ assay, respectively.	No API, low or higher APIs, other APIs, expired medicines

API = active pharmaceutical ingredients; HPLC = high-performance liquid chromatography; HPLC-UV-PDA = high-performance liquid chromatography with ultraviolet photo-diode array detection; NIR = near infrared spectrometry; Sn = senstivity; Sp = specificity; TLC = thin-layer chromatography.
